# Adjunctive use of a concentrated phytodietary compound in the management of prolonged diarrhea in infants: evidence from a real-world study

**DOI:** 10.3389/fped.2026.1699488

**Published:** 2026-02-17

**Authors:** Wen-Li Yang, Yuan-Da Zhang, Jing-Jing Cao, Hong-Mei Huang, Wen-Li Zhao, Jie Yan

**Affiliations:** 1 Department of Clinical Nutrition, Beijing Children’s Hospital, Capital Medical University, National Center for Children’s Health, Beijing, China; 2 Department of Nutrition, Baoding Hospital of Beijing Children’s Hospital, Capital Medical University, Regional Center for Children’s Health, Baoding, Hebei, China; 3 Department of Laboratory Medicine, Beijing Tongren Hospital, Capital Medical University, Beijing, China

**Keywords:** concentrated compound phytodietary supplement, infants, malnutrition, prolonged diarrhea, real-world study

## Abstract

**Objective:**

The aim of this study was to evaluate the adjunctive therapeutic efficacy and safety of a concentrated compound phytodietary supplement in the management of prolonged diarrhea in infants, using data derived from real-world clinical settings.

**Methods:**

This retrospective, observational study analyzed real-world clinical data from three tertiary hospitals. Medical records of infants aged 6–12 months diagnosed with prolonged diarrhea (duration >14 days) between January 2020 and August 2021 were reviewed. Infants who received standard therapy alone were assigned to the control group, while those who received standard therapy in combination with the phytodietary supplement comprised the intervention group. Therapeutic response was assessed after 7 days of treatment and categorized as cure, improvement, or ineffective.

**Results:**

A total of 505 infants met the inclusion criteria, with 242 infants in the intervention group and 263 in the control group. Baseline characteristics, including sex distribution, median age, and nutritional status, did not differ significantly between groups. At day 7, the intervention group demonstrated cure, improvement, and ineffective rates of 52.9%, 40.9%, and 6.2%, respectively, yielding an overall efficacy rate of 93.8%. In contrast, the control group exhibited rates of 10.3%, 63.9%, and 25.8%, respectively, with a total efficacy rate of 74.2% (*χ*^2^ = 33.621, *p* < 0.01). The median diarrhea duration was 4 days in the intervention group vs. 6 days in the control group. Among 133 infants in the intervention group who underwent extended follow-up, the baseline prevalence of malnutrition was 40.6%. After 28 days, the number of complementary food categories increased by more than four, the prevalence of malnutrition declined to 31.6%, and the median Z-score for anthropometric indicators increased by 0.2 relative to baseline (*p* < 0.001, Cohen's *d* = 0.66).

**Conclusion:**

This real-world analysis suggests that the adjunctive use of a concentrated phytodietary supplement in infants with prolonged diarrhea may enhance therapeutic outcomes, including a significant reduction in diarrhea duration and improved nutritional status. Continued use following resolution of diarrhea may support the introduction of complementary foods and contribute to improved growth trajectories. These findings support the potential clinical utility of phytodietary supplementation as an adjunct to standard care in the pediatric population.

## 1 Introduction

Globally, an estimated 1.7 billion episodes of diarrhea occur annually among children under the age of 5, with each child experiencing an average of 2–3 episodes per year. Diarrhea is the second leading cause of mortality in this age group (1). Among these cases, prolonged diarrhea, defined as diarrhea lasting more than 14 days, accounts for approximately 3%–20% of all diarrheal episodes in young children and is associated with an increased risk of dehydration, malnutrition, and hospitalization when not promptly or adequately managed (2).

Although current first-line treatments for pediatric diarrhea, such as oral rehydration salts, diosmectite, and probiotic formulations, are effective in many cases, their efficacy may be limited in prolonged diarrhea. Additionally, challenges related to adherence and symptom persistence are frequently reported in real-world clinical practice.

Emerging evidence suggests that certain components found in concentrated phytodietary supplements, including taro (*Colocasia esculenta*), carrot (*Daucus carota* subsp. *sativus*), glutinous rice, and rice (*Oryza sativa*), may contribute to the management of diarrhea through mechanisms involving modulation of intestinal inflammation, immune function, and gut microbiota composition (3–7). However, most existing studies have been conducted in controlled settings, and evidence from real-world clinical environments remains scarce.

This study aimed to evaluate the adjunctive therapeutic efficacy and safety of a concentrated compound phytodietary supplement in infants diagnosed with prolonged diarrhea, with particular emphasis on its potential to reduce diarrhea duration, using real-world clinical data.

## 2 Materials and methods

### 2.1 General data

This study employed a retrospective, observational design based on real-world clinical data. Medical records of infants diagnosed with prolonged diarrhea who presented to Beijing Children's Hospital, Capital Medical University; Beijing Tongren Hospital, Capital Medical University; and Baoding Hospital, Beijing Children's Hospital, between 16 January 2020 and 6 August 2021 were reviewed.

Baseline information was extracted, including demographic characteristics, anthropometric measurements, feeding history, allergy history, stool characteristics and frequency, routine stool examination results, and findings from etiological testing.

### 2.2 Study groups

#### 2.2.1 Inclusion criteria

Infants were eligible for inclusion if they met all of the following criteria:

Aged between 6 and 12 months, irrespective of sex
Diagnosed with prolonged diarrhea (diarrhea lasting >14 days)
Mild to moderate diarrhea severity
Stool leukocyte count <10 per high-power field on microscopic examination
Absence of toxic symptoms, including moderate to severe dehydration, shock, or altered consciousness

#### 2.2.2 Exclusion criteria

Infants were excluded if any of the following conditions were present:

Infectious diarrhea caused by identified pathogens (e.g., bacteria, parasites) or cytomegalovirus enteritis
Use of antimicrobial agents within the preceding two weeks
Diagnosed with coeliac disease, inflammatory bowel disease, or protein-losing enteropathy
Presence of comorbidities, including malignancy, hematologic disorders, immunodeficiency, or severe systemic infections

#### 2.2.3 Intervention group

Infants meeting the above inclusion and exclusion criteria were assigned to the intervention group if they also satisfied the following conditions:

Informed consent was obtained from the legal guardian, and the concentrated compound phytodietary supplement was administered as prescribed. Both healthcare providers and guardians were informed of the study protocol
No known hypersensitivity to any components of the supplement (taro, carrot, glutinous rice, or rice)
Completion of required follow-up and provision of outcome data

#### 2.2.4 Control group

Infants who met the inclusion and exclusion criteria during the same study period but did not receive the phytodietary supplement were assigned to the control group.

### 2.3 Treatment methods

#### 2.3.1 Control group

Infants in the control group received conventional treatment tailored to their clinical condition, which included oral rehydration solution (ORS), probiotics, diosmectite, and dietary modifications, as per standard care guidelines.

#### 2.3.2 Intervention group

Infants in the intervention group received the same conventional treatment in addition to a concentrated compound phytodietary supplement. The supplement is a powdered formulation composed of taro, carrot, glutinous rice, and rice, produced using proprietary processing technology. The product is patented and approved for use in infants aged 4 months and older.

Each serving contains 7 g of powder. The prescribed dosage ranged from 3.5 g to 7 g per administration and was given orally two to three times daily after mixing with warm water to form a paste. In cases of dry stools or difficulty with defecation, the daily dosage was reduced by 3.5 g. If no recurrence of loose stools or increased stool frequency occurred, the dosage was gradually tapered until discontinuation.

### 2.4 Data collection

#### 2.4.1 Intervention group

Data were collected through caregiver self-reporting using a secure WeChat-based mini-program. Caregivers recorded stool frequency, stool characteristics, relevant dietary changes, and any potential adverse events. Daily reporting was required until resolution of diarrheal symptoms, with additional mandatory entries on days 3, 5, and 7 following treatment initiation. Each report included dietary information and the presence or absence of any adverse symptoms.

#### 2.4.2 Control group

Data were retrospectively extracted from contemporaneous medical records, including documentation from the initial outpatient consultation and the follow-up evaluation on day 7.

### 2.5 Definitions and efficacy criteria

Diarrhea was defined as a change in stool characteristics and/or an increase in stool frequency relative to the infant's baseline, with altered stool characteristics considered the primary diagnostic criterion. Alterations included loose, watery, mucoid, or purulent bloody stools, corresponding to type 6 or type 7 on the Bristol Stool Form Scale. Prolonged diarrhea was defined as diarrhea persisting for a duration of 2 weeks to 2 months (8).

Moderate diarrhea was defined as approximately 10 bowel movements per day, or a marked increase compared with baseline frequency, with loose or watery stools corresponding to Bristol Stool Form Scale types 6–7. Severe diarrhea was defined as 8–15 bowel movements per day, predominantly watery stools corresponding to Bristol Stool Form Scale type 7, with large stool volume and an acidic or foul odor, and occasionally accompanied by mucus or small amounts of blood.

Anthropometric assessments were conducted in accordance with the World Health Organization (WHO) Child Growth Standards for children aged 0–5 years (https://www.who.int/tools/child-growth-standards/standards). Z-scores for weight-for-age, length-for-age, and weight-for-length were calculated for each infant. Nutritional status was classified as follows:

Mild malnutrition: Z-score between −2 and −1
Moderate malnutrition: Z-score between −3 and −2
Severe malnutrition: Z-score ≤ −3

The incidence of malnutrition was calculated as the proportion of infants with Z-scores in the mild, moderate, or severe categories: (number of mild + moderate + severe malnutrition cases)/total number of cases.

The lowest Z-score among weight-for-age, length-for-age, and weight-for-length was used to determine overall nutritional status.

Efficacy status:

Cured: Stool frequency and characteristics returned to normal (defined as Bristol Stool Scale types 2–5) within 7 days of treatment initiation.
Effective: Improvement in stool frequency and/or stool characteristics within 7 days, sustained for more than 24 h. Improvement in stool characteristics was defined as a reduction of at least one category on the Bristol Stool Scale.
Ineffective: No improvement or worsening of stool frequency and characteristics after 7 days (9).

The total efficacy rate was calculated as the sum of the cure rate and improvement rate.

### 2.6 Statistical methods

Statistical analyses were performed using SPSS software, version 22.0 (IBM Corp., Armonk, NY, USA). Categorical variables are presented as frequencies and percentages. Between-group comparisons for categorical data were conducted using the chi-squared (*χ*^2^) test. Ninety-five percent confidence intervals (95% CIs) were calculated using the Wald method.

The distribution of continuous variables was assessed using the Shapiro–Wilk test. Normally distributed data are presented as mean ± standard deviation (SD). For between-group comparisons of normally distributed variables, independent-sample *t*-tests were used; paired *t*-tests were applied for within-group comparisons. Non-normally distributed data are reported as medians, and between-group comparisons were performed using the Wilcoxon rank-sum test.

A *p* value of <0.05 was considered statistically significant. No formal correction for multiple comparisons was applied, as all analyses were exploratory. There were no missing or incomplete data.

## 3 Results and analysis

### 3.1 Baseline characteristics and study population

The sample size was determined by the number of eligible cases available during the study period. A total of 505 infants diagnosed with prolonged diarrhea were included in the analysis. The intervention (*n* = 242) and control (*n* = 263) groups were well-balanced at baseline (Table 1). A *post-hoc* power analysis confirmed the study had >99% power (*α* = 0.05) to detect the observed difference in efficacy rates, validating the sample size adequacy.

**Table 1:** Baseline characteristics of infants aged 6 to 12 months with prolonged diarrhea.

Clinical characteristics	Intervention group	Control group	*P*-value
Number of cases	242	263	–
Male, *n* (%)	123 (50.8)	138 (52.5)	0.130
Female, *n* (%)	119 (49.2)	125 (47.5)	0.085
Median age, months	9 (6–12)	8.8 (6–12)	0.592
Malnutrition, *n* (%)	93 (38.4)	102 (38.8)	0.226
Mild malnutrition, *n* (%)	63 (67.7)	70 (68.6)	0.125
Moderate malnutrition, *n* (%)	26 (28.0)	27 (26.5)	0.061
Severe malnutrition, *n* (%)	4 (4.3)	5 (4.9)	0.383

### 3.2 Primary efficacy: diarrhea resolution and symptom improvement

**Figure 1:**
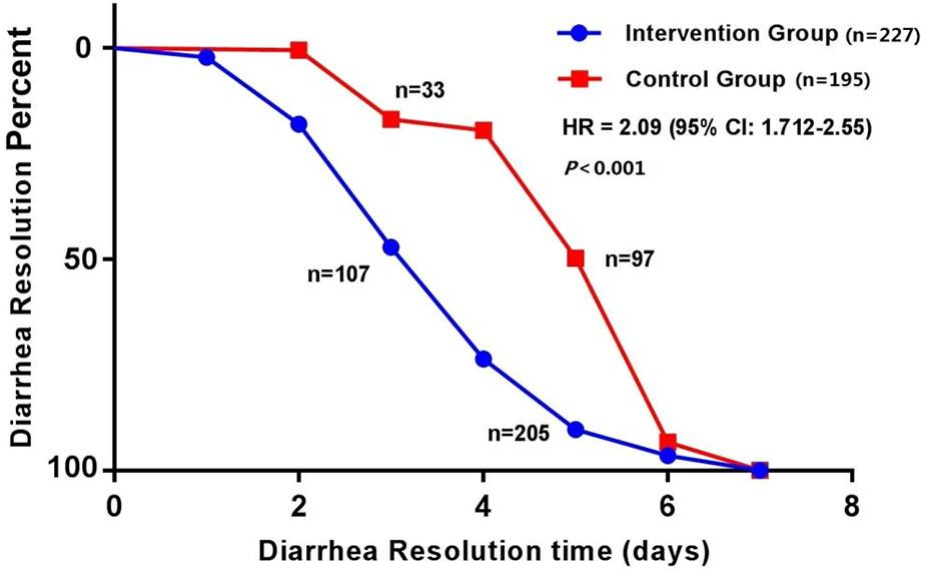
Kaplan–Meier survival curves for diarrhea resolution in the two groups.

The adjunctive use of the phytodietary supplement was associated with a significantly superior therapeutic outcome. In the intervention group, the cure, effective, and ineffective rates were 52.9% (128 cases), 40.9% (99 cases), and 6.2% (15 cases), respectively, resulting in a total efficacy rate of 93.8% (227 cases) [95% CI 86.7%, 99.8%]. In the control group, the corresponding rates were 10.3% (27 cases), 63.9% (168 cases), and 25.8% (68 cases), yielding a total efficacy rate of 74.2% (195 cases) [95% CI 68.7%, 79.5%]. The total efficacy rate was significantly higher in the intervention group (*χ*^2^ = 33.621, *p* < 0.01). A critical finding was the accelerated time to recovery. The median duration of diarrhea was 4 days in the intervention group, which was significantly shorter than the 6 days observed in the control group (t = −2.32, *p* < 0.01). In the intervention group, diarrhea resolved within 3 days in 107 infants (47.2%) and within 5 days in 205 infants (90.3%). In the observation group, corresponding resolution rates were 33 infants (12.5%) within 3 days and 97 infants (36.9%) within 5 days. Cumulative resolution rates were consistently and significantly higher in the intervention group at all assessed time points (Figure 1). This absolute difference of 30% corresponds to a Number Needed to Treat of approximately 4 (95% CI: 2.4, 5.7). The Relative Risk for treatment success was 2.5 (95% CI: 1.61, 3.87). The median daily dose of the supplement administered was 10.5 g, with no clear dose-response relationship identified within the prescribed dosage range (3.5 g–21 g per day).

Furthermore, a subgroup analysis revealed no significant interaction effects (p for interaction >0.1) based on sex or baseline nutritional status, suggesting the treatment effect was consistent across these demographic and clinical factors.

### 3.3 Exploratory analysis: nutritional recovery and complementary feeding

In the intervention group, a substantial proportion of infants with prolonged diarrhea had previously discontinued complementary feeding or were receiving only limited carbohydrate-based complementary foods. Following improvement of diarrheal symptoms and under physician guidance, the concentrated compound phytodietary supplement was continued, and complementary foods were gradually reintroduced.

A total of 133 infants in the intervention group were followed up for 28 days. During this period, the number of complementary food categories introduced increased by more than 4, with a maximum of 11 new food types. Newly introduced complementary foods included scorched rice flour, carrot, lotus root, lotus seed, purple sweet potato, pumpkin, apple, bok choy, pork liver, and beef.

**Figure 2:**
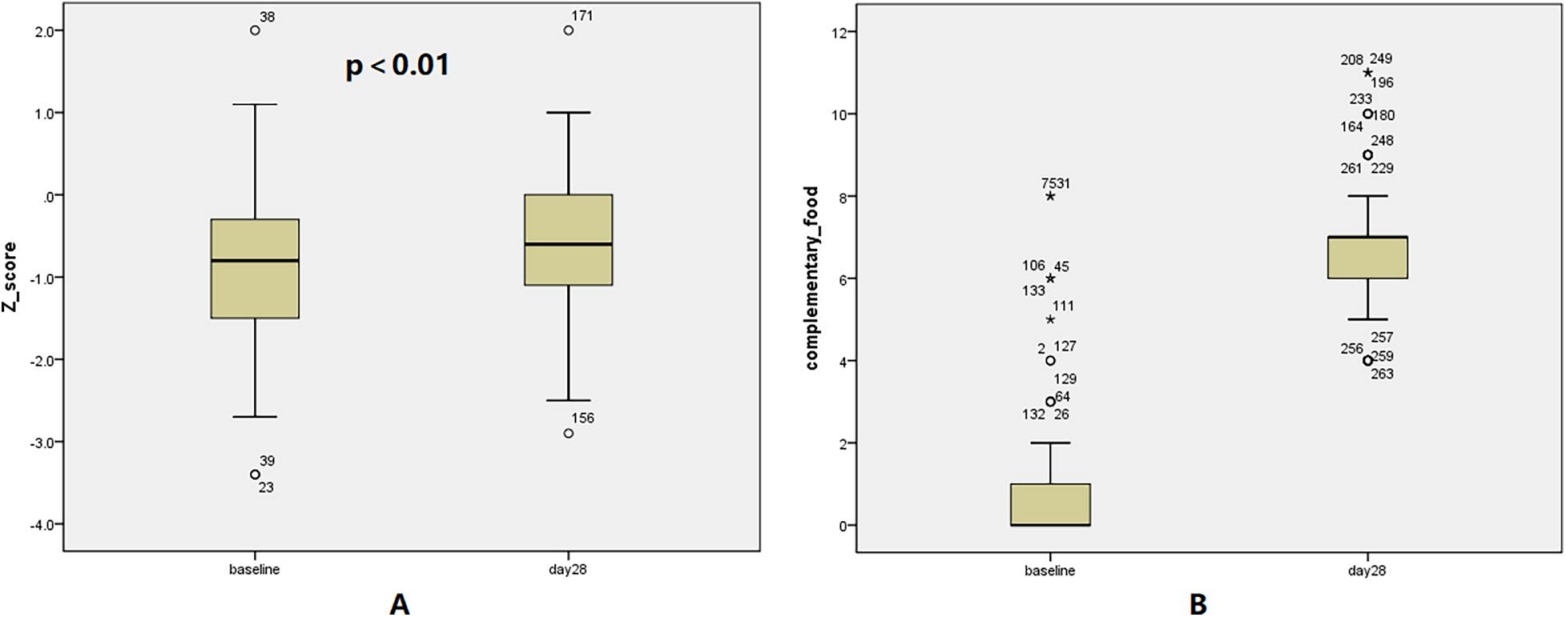
Distribution of Z-scores and number of complementary food categories. The distribution of Z-scores **(A)** and the number of complementary food categories **(B)** at baseline and day 28.

At baseline, malnutrition (Z-score <−1) was prevalent, affecting 40.6% (54/133) of this sub-cohort. After 28 days of continued supplementation alongside guided reintroduction of complementary foods, the prevalence of malnutrition decreased to 31.6% (42/133), with the most notable reduction seen in the moderate and severe categories. 33.3% (12/36) of mildly malnourished infants reverting to normal status, and 62.5% (10/16) of moderately malnourished infants improving to the mild category. Both severely malnourished infants improved to a moderate status. Critically, no infant experienced a deterioration in nutritional status (Table 2). Concurrently, the mean Z-score for anthropometric indicators increased from −0.84 ± 0.39 to −0.61 ± 0.31. The median change in Z-score was 0.2 (range: 0.2–0.8), and this improvement was statistically significant with a medium effect size (*p* < 0.001, Cohen's d = 0.66), indicating a clinically meaningful advancement in growth trajectories. The distribution of changes in complementary food types and anthropometric Z-scores in infants before and after the 28-day follow-up is shown in Figure 2.

**Table 2:** Anthropometric recovery and Z-score dynamics.

Nutritional parameter	Baseline (Day 0)	Follow-up (Day 28)	Mean change (*Δ*)	*P*-value
Anthropometrics, Z-score, (Mean ± SD)	−0.84 ± 0.39	−0.61 ± 0.31	0.2	<0.01
Weight-for-Age Z-score (WAZ), (Mean ± SD)	−0.87 ± 0.41	−0.53 ± 0.37	0.3	<0.01
Weight (kg), (Mean ± SD)	7.9 ± 1.10	8.7 ± 0.95	1.0	<0.05
Malnutrition Severity, *n* (%)	54 (40.6)	42 (31.6)	12 (9)	0.08
Mild, *n* (%)	36 (27.1)	34 (25.6)	4 (1.5)	<0.05
Moderate, *n* (%)	16 (12.0)	8 (6.0)	8 (6.0)	<0.05
Severe, *n* (%)	2 (1.5)	0	2 (1.5)	<0.05

### 3.4 Adverse reactions

**Figure 3:**
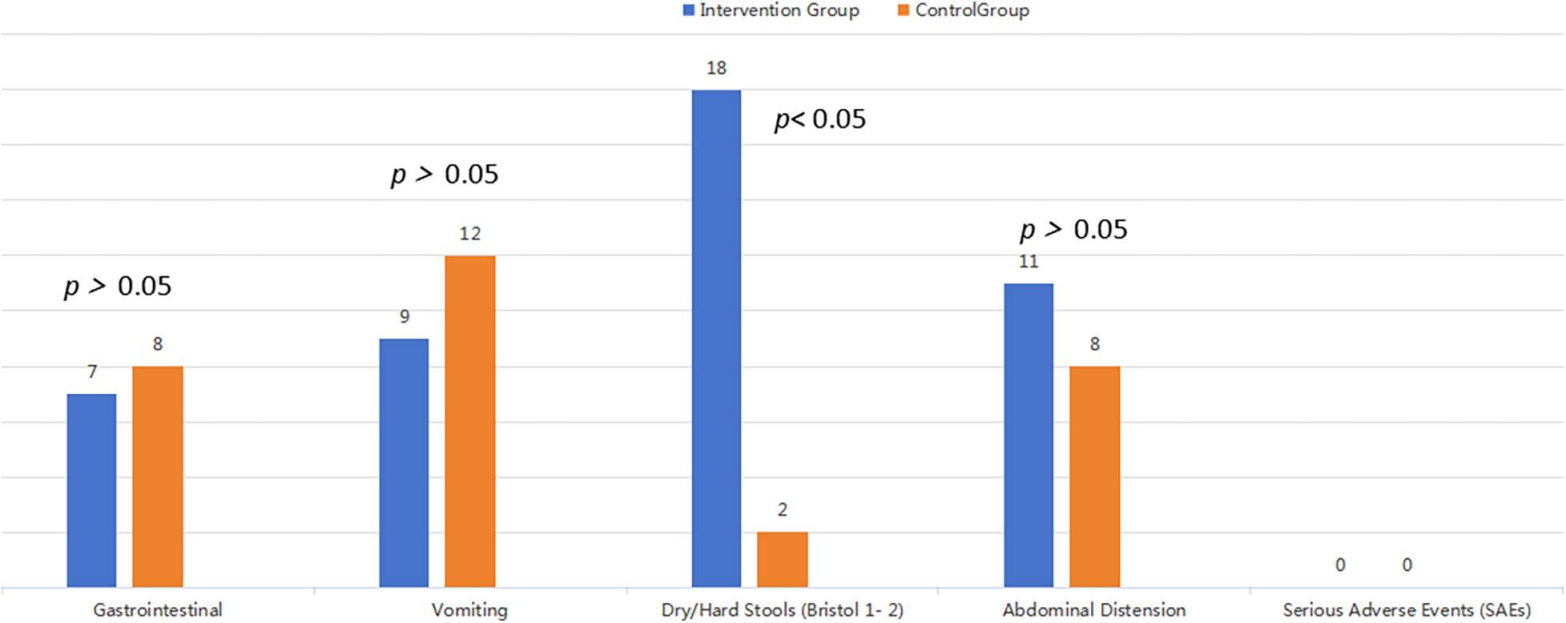
Bar chart of adverse events.

The safety analysis revealed a generally comparable adverse event profile between the intervention and control groups for most categories. The incidence of gastrointestinal AEs (Intervention: 7 vs. Control: 8), vomiting (9 vs. 12), and abdominal distension (11 vs. 8) was not significantly different between the two groups (Figure 3). A small number of infants in the intervention group (*n* = 18) experienced dose-related adverse events, specifically dry stools and difficult defecation (Bristol types 1–2). These gastrointestinal symptoms were manageable and resolved promptly upon dose reduction or discontinuation, indicating that the supplement's effects on stool consistency are dose-dependent and reversible. No serious adverse events or recurrence of diarrhea linked to the supplement were reported during the follow-up period.

## 4 Discussion

Findings from this real-world study indicate that the adjunctive use of a concentrated compound phytodietary supplement, in combination with conventional therapy, was associated with a 2-day reduction in the duration of diarrhea and a 19.6% improvement in recovery rates compared to standard care alone in infants with prolonged diarrhea.

Prolonged diarrhea in infants is primarily attributed to persistent infections; however, noninfectious etiologies, such as lactose intolerance, deficiency of osmotic regulators, postinfectious irritable bowel syndrome, and functional gastrointestinal disorders, may also contribute. It remains a significant risk factor for malnutrition in infants and young children and is associated with elevated rates of infant and child mortality in low-resource settings (WHO) (1).

The supplement evaluated in this study, composed of taro, carrot, glutinous rice, and rice, contains bioactive plant-derived constituents. These ingredients, prepared through specialized formulation and food-processing techniques, are considered natural dietary substances with a long-standing history of use in the dietary management of diarrhea. The improved clinical outcomes observed in the intervention group suggest that the phytodietary supplement may have contributed to a more rapid resolution of diarrheal symptoms. However, causality cannot be established in the absence of a randomized controlled design.

Taro (*Colocasia esculenta*, family Araceae) has been traditionally used in the management of diarrhea, digestive disorders, and food allergies in infants and young children. Several studies have reported a lower incidence of diarrhea among infants consuming taro-based complementary foods compared to those fed rice- or wheat-based products such as bread (3–5, 10, 11).

Phytochemical analyses have identified bioactive constituents in taro, including decanoic acid, pentadecanoic acid, *n*-hexadecanoic acid, lipoxygenase, and various secondary metabolites with demonstrated antibacterial properties. *In vitro* studies have shown that crude taro extracts inhibit the growth of several pathogenic microorganisms, including *Pseudomonas aeruginosa*, *Serratia* spp., *Escherichia coli*, *Salmonella*, *Shigella*, *Enterococcus*, *Aspergillus*, and *Candida albicans* (3).

In addition, taro-derived compounds such as saponins, terpenoids, flavonoids, tannins, and steroidal alkaloids have been reported to exert anti-inflammatory effects, which may further contribute to its therapeutic potential in gastrointestinal disorders (3).

Carrots (*Daucus carota* subsp. *sativus*) are a source of acidic oligosaccharides that inhibit the adhesion of multiple pathogenic microorganisms to HEp-2 epithelial cells and human intestinal mucosa, thereby reducing colonization and risk of enteric infection (6). Since the first report by Moro in 1908 on the therapeutic use of carrots for infant diarrhea in Germany, carrots have remained a key component in the dietary management of pediatric diarrhea (6).

Clinical studies have demonstrated that ORS supplemented with carrot and rice reduce both the duration of diarrhea and stool volume in infants aged 3 months to 2 years with mild to moderate dehydration, while maintaining a favorable safety profile (6, 12, 13). In addition to their anti-adhesive effects, carrot-derived oligosaccharides exhibit prebiotic activity by modulating gut microbiota composition and influencing immune and inflammatory responses.

Recent studies have highlighted the bidirectional relationship between prolonged diarrhea and intestinal dysbiosis (9). Notably, increased bifidobacterial abundance has been observed in infants who consumed carrots during diarrheal episodes, suggesting a role in restoring microbial balance and supporting intestinal health (14).

Glutinous rice is a source of protein, complex carbohydrates, calcium, phosphorus, iron, B vitamins, and starch. In traditional Chinese medicine, it has been historically used for gastrointestinal support. Ancient texts such as the *Ming Yi Bie Lu* (Records of Famous Physicians) describe glutinous rice as strengthening the middle burner, generating warmth, and producing firmer stools (15). Similarly, the *Ben Cao Gang Mu* (Compendium of Materia Medica) records its role in supporting spleen and stomach function, reducing cold-induced diarrhea, and decreasing stool volume (16).

In the formulation of the concentrated compound phytodietary supplement used in this study, a physical gelatinization process was applied to convert long-chain starches into short-chain starches. This method improves digestibility without the use of chemical additives, ensuring safety and suitability for infant consumption.

Diarrhea is a major risk factor for malnutrition, and the two conditions often perpetuate one another in a cyclical relationship. Nutritional recovery following diarrheal episodes typically occurs in stages, as intestinal mucosal repair requires approximately 2–6 weeks (17). Continued supplementation during this period may offer anti-inflammatory effects, support mucosal integrity, provide carbohydrate-based energy, and promote nutrient absorption. It may also facilitate the reintroduction of complementary foods in a gradual and nutritionally balanced manner.

In this study, infants who continued supplementation following the resolution of diarrhea exhibited improvements in anthropometric measures, with a median Z-score increase of 0.2, consistent with early-stage nutritional recovery.

Multiple traditional foods have been utilized in the nutritional management of prolonged diarrhea, including milk (with or without lactose), soy protein, hydrolyzed proteins, and amino acids (18). However, a standardized nutritional treatment protocol has not yet been established. A multicenter study conducted by the WHO in several countries provided simplified clinical guidelines and demonstrated the successful management of persistent diarrhea using locally available foods (19). Nevertheless, no universally effective dietary regimen has been identified across diverse geographic and socioeconomic contexts.

The concentrated compound phytodietary supplement evaluated in this study incorporates four food-based components that undergo physical processing to convert long-chain starches into short-chain forms. This modification may enhance digestibility and energy availability, potentially contributing to a shortened duration of diarrhea when used in conjunction with conventional therapy.

Several limitations of this study should be acknowledged. First, data were obtained from medical records and caregiver-reported entries via digital platforms, which may introduce reporting bias and interindividual variability. Second, the non-blinded design increases the risk of both observer and participant bias. Third, the absence of a placebo control limits the ability to distinguish true treatment effects from placebo responses or concurrent clinical care. Additionally, the follow-up duration was relatively short, and incomplete follow-up data in some cases limited the assessment of longer-term outcomes. The sample size, while adequate for exploratory analysis, was relatively small and may contribute to selection bias.

Despite these limitations, the real-world setting of this study and inclusion of infants with malnutrition enhance the clinical applicability of the findings. Real-world studies provide important insights into the effectiveness and safety of interventions under routine care conditions and may help inform clinical decision-making (20). While the results are encouraging, they should be interpreted with caution given the observational study design. Future randomized controlled trials with larger, more diverse populations are warranted to confirm these findings. Additional research could also explore the supplement's effects on gut microbiota composition and evaluate its potential utility in older pediatric populations.

## 5 Conclusion

The adjunctive use of a concentrated compound phytodietary supplement was associated with accelerated symptom improvement and favorable tolerability in infants aged 6–12 months with prolonged diarrhea. No significant adverse effects were observed during the study period. Additionally, continued supplementation appeared to support improvements in nutritional status and growth indicators.

These preliminary findings, derived from a real-world setting, suggest potential clinical utility for this phytodietary intervention. However, confirmation through larger, well-controlled randomized trials is necessary to validate efficacy, assess long-term outcomes, and further elucidate mechanisms of action.

## Data Availability

The original contributions presented in the study are included in the article/Supplementary Material, further inquiries can be directed to the corresponding author.
